# Andrographolide as a Therapeutic Agent Against Breast and Ovarian Cancers

**DOI:** 10.1515/biol-2019-0052

**Published:** 2019-12-04

**Authors:** Swarna Latha Beesetti, Mavuluri Jayadev, Gnana Veera Subhashini, Lamjed Mansour, Saleh Alwasel, Abdel Halim Harrath

**Affiliations:** 1 P.O. Box 2455, Department of Zoology, College of Science, King Saud University 11451 Riyadh- Saudi Arabia; 2 Baba Clinical and Genomic Research Centre, Ticel Biopark, Taramani, Chennai 600113, Tamil Nadu, India

**Keywords:** Andrographolide, MMP inhibitor, apoptosis, ovarian cancer, breast cancer

## Abstract

Andrographolide (ANDR), isolated from *Andrographis paniculata*, is a medicinal compound effective against infections, inflammatory disorders, and various cancers. In the present study, the effects of ANDR on NFkB (nuclear factor kappa-light-chain-enhancer of activated B cells) activation, caspase-8-mediated apoptosis and pyroptosis, and extra cellular matrix (ECM) degradation were analyzed in A431, MDA-MB231, and SKOV-3 cell lines. Results showed that ANDR inhibited the growth and proliferation of cancer cell lines by inhibiting NFkB signaling. A significant decrease in phospho-p65 level was observed upon increasing ANDR concentration in epidermoid carcinoma and breast cancer cell lines, A431 and MDA-MB231, respectively. Accordingly, upon ANDR treatment, the expression of caspase-8 was increased, whereas no significant induction of caspase-1 expression was observed. Moreover, we observed a significant increase in the expression of tissue inhibitor of metallopeptidase-1 (TIMP1) upon increasing ANDR concentration. Such induction of TIMP1 inhibited the activity of matrix metallopeptidase-7 (MMP-7), thus preventing the degradation of ECM. Therefore, as ANDR shows cytotoxicity towards cancer cells via the NFkB signal transduction pathway without inducing pyroptosis and blocks breast and ovarian cancer invasion by inhibiting MMP-7 expression through TIMP1 up-regulation, it has the potential to be developed as a drug targeting ovarian and breast cancers.

## 1 Introduction

Ovarian and breast cancers are two of the major causes of mortality in women [1, 2, 3]. The rate of recurrence of these cancers is high because of the early onset of occurrence and resistance mechanism to treatment [4]. The use of chemotherapy has decelerated cancer progression to some extent. However, emerging literature shows that cancer cells have gained resistance to the available drugs [5, 6]. Chemical modification of the existing natural anticancer drugs has increased the efficiency of the drugs; however, it has resulted in unexpected side effects, such as renal toxicity, nausea, neurotoxicity, and vomiting [7]. Drugs such as cyclophosphamide, cisplatin, docetaxel, doxorubicin, and paclitaxel are widely used to treat various cancers [8]. Furthermore, these drugs were used in combination to increase specificity and efficiency, although the combinatorial approach remained relatively ineffective because of emerging multi-drug resistance. Hence, it is essential to explore the signaling pathways and genes affected by the existing novel drugs. A new strategy for improved treatment might be the careful choice of the most appropriate drugs used in combination in order to have a specific target.

Various natural products have been successfully employed to treat ovarian and breast cancers [9, 10, 11]. In particular, andrographolide (ANDR), derived from *Andrographis paniculata*, is a bioactive herbal medicine with several applications. It has been reported to have antioxidant, anticancer, antibacterial, antifungal, antiviral, including dengue and human immunodeficiency (HIV) viruses, antihyperglycemic, anti-platelet aggregation, antidiabetic properties [12, 13, 14, 15, 16, 17, 18]. ANDR also is used widely in the treatment of diarrhea, fever, and inflammation, as well to treat many infectious diseases [19]. ANDR is also reported to be an effective immune stimulator and has a scavenging and protective effect against hepatic lipid peroxidation [20, 21]. *In vitro* and *in vivo* studies showed its cytotoxic effect on cell cycle progression, apoptosis, cell invasion, migration, and angiogenesis [22, 23, 24].

As cancer cells develop multi-drug resistance [25], new drugs are being synthesized by modifying the present drugs to make them highly competent and effective against the genes involved in cancer initiation and progression. Natural anticancer drugs derived from plants are considered to be very effective with limited side effects [26]. The present study focused on the effect of ANDR on the functions of A431, MDA-MB231 and SKOV-3 cell lines. Notably, we sought to unravel the role of the MMP inhibitor and the NFkB-FLIP-caspase-8 signal in ANDR mediated anticancer effect in ovarian and breast cancers; which has not been reported to date according to our knowledge.

## 2 Materials and methods

### 2.1 Materials

ANDR (>98% purity, TCI Chemicals Pvt. Ltd., India) was dissolved in dimethyl sulfoxide (DMSO) to prepare a stock solution (10 mM) and stored at room temperature. Fetal bovine serum (FBS), phosphate buffer solution (PBS), penicillin, Dulbecco’s modified eagle medium (DMEM), and trypsin-ethylenediaminetetraacetic acid (EDTA) were procured from HiMedia Laboratory (P) Ltd., India. A protease and phosphatase inhibitor cocktail was obtained from Pierce (Rockford, IL) and the antibodies against phospho-p-65, p-65, and vinculin were obtained from Cell Signaling Technology, USA. Caspase-8, caspase-1, MMP-7, and TIMP-1 antibodies were obtained from Cistron Biolabs, India. Secondary mouse and rabbit antibodies conjugated with horseradish peroxidase were obtained from Santa Cruz (TX, USA). Enhanced chemiluminescence solution was obtained from GE Healthcare (Chicago, USA).

### 2.2 Methods

#### 2.2.1 Cell lines and cell culture

MDA-MB-231 human breast cancer cell line, SKOV-3 human ovarian cancer cell line, and A431 epidermoid carcinoma cell line were obtained from National Centre for Cell Sciences (NCCS, Pune, India). The cell lines were cultured in high glucose (4.5 g/L) DMEM supplemented with 10% FBS, 100 mg/mL streptomycin, and 100 units/mL penicillin at 37 °C under 5% CO_2_ and 95% air. The cells were harvested from the logarithmic phase of cultures and the media was replaced once every two days.

#### 2.2.2 Andrographolide (ANDR) treatment

A stock solution (10 mM) of ANDR was prepared in DMSO, and further dilutions were made in the cell culture media. The inhibitory concentration of ANDR on cancer cell lines was determined from available literature (Supplementary Table 1), and the cells were treated with 10 μM, 20 μM or 50 μM of ANDR for 4 h [27].

#### 2.2.3 Protein isolation, SDS-PAGE, and western blotting assay

Cancer cells were seeded in 100 mm cell culture dishes and left overnight for the cells to attach. After 24 hours, the cells were treated with 10 μM, 20 μM, and 50 μM of0 ANDR for 4 h. For the isolation of whole cell protein, radioimmunoprecipitation assay (RIPA) buffer containing 50 mM Tris-HCl (pH 7.4), 1% Triton X-100, 150 mM NaCl, 0.5% deoxycholate, 0.1% sodium dodecyl sulfate (SDS), and the protease/phosphatase inhibitor cocktail was used. The concentration of proteins was measured spectrophotometrically using a Bio-Rad protein estimation kit (NJ, USA). Equal amounts of protein lysates were separated by 8% SDS polyacrylamide gel electrophoresis and transferred to the nitrocellulose membrane. The membranes were blocked with 5% BSA (Bovine Serum Albumin) for 1 h, and then incubated overnight at 4°C with the desired primary antibodies, followed by incubation with the respective secondary antibody for 1 h. The proteins were visualized using an Enhanced ChemiLuminiscence kit (GE Healthcare, Chicago, USA).

#### 2.2.4 ELISA (Enzyme-linked immunosorbent assay)

MDA-MB 231 and SKOV-3 cell lysates were collected after ANDR treatment. Standards and analytes were diluted with coating buffer and added to the wells. After 2 h of incubation, the wells were washed with wash buffer and incubated for 1 h with blocking solution at room temperature. After three washes, the analytes were incubated with primary antibodies against MMP-7/TIMP-1/caspase-8/caspase-1 for 2 h, followed by incubation with the respective secondary antibody for 1 h with intermittent washing. TMB (Tetramethylbenzidine) substrate (100 μL/well) (Abcam, Cambridge, USA) was added and the colorimetric reaction was stopped after 10 min by adding the stop solution (100 μL/well). The readings were recorded at an absorbance of 450 nm using spectrophotometer.

#### 2.2.5 Statistical analysis

Each experiment was performed three times. GraphPad PRISM was used for the statistical analysis. Statistical significance was evaluated by calculating the *P*-values. Differences at *P* <0.05 were considered statistically significant.

## 3 Results and discussion

### 3.1 Effect of ANDR on NFkB activation in breast and epidermoid carcinoma cell lines

**Fig. 1:**
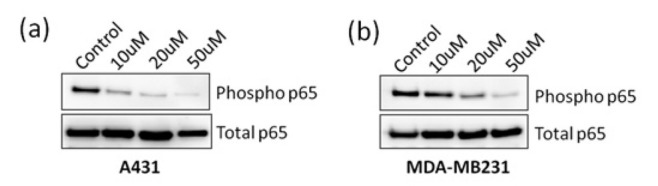
Western blot analysis of phospho-p65 and total p65 levels in A431 (a) and MDA-MB231 (b) cell lines upon treatment with increasing concentrations of ANDR. Treatment was for 4 h after which the lysates were prepared.

As ANDR was observed to affect tumor development, we examined the levels of total p65 and its phosphorylation status upon ANDR treatment by western blot analysis. Results showed a decrease in phospho-p65 levels with increasing concentrations of ANDR in epidermoid carcinoma and breast cancer cell lines, A431 and MDA-MB231, respectively (Fig. 1a and b). Therefore, ANDR inhibits the growth and proliferation of cancer cell lines by inhibiting NFkB signaling.

ANDR has multiple targets at the membrane, cytoplasm, and nuclear levels, which in turn affected the targets in our study directly or indirectly [28]. The transcription factor NFkB is a key molecule that regulates various cellular processes within cancer cells. It promotes tumor growth, proliferation, epithelial mesenchymal transition, metastasis, and angiogenesis [29]. It is composed of two subunits, p50 and p65 [30], and is highly expressed and activated in a wide variety of cancers. In fact, cancer cells perform most of their functions by activating this vital transcription factor. The activation of NFkB is the primary event in response to certain stimuli such as stress, and viral and bacterial infections [31]. It mediates several cellular functions, such as cell survival, proliferation, cytoskeleton remodeling, epithelial to mesenchymal transition, inflammation, and defense mechanisms [29, 32]. The results of the present study revealed the inhibition of p65 activation as a response to ANDR treatment, which ultimately inhibited cancer progression. These observations are in corroboration with the previous findings [33]. In fact, ANDR is known to inhibit NFkB signaling in vascular smooth muscle cells and lung cancer cell lines [34]. We can conclude that ANDR inhibits cancer cell proliferation via inhibition of NFkB signal transduction pathway. To the best of our knowledge, this is the first study that investigated the effect of ANDR treatment on the metastatic breast cancer cell line MDA-MB-231, ovarian cancer cell line SKOV-3, and epidermoid carcinoma cells A431.

### 3.2 Effect of ANDR on apoptosis and pyroptosis in breast and ovarian cancers

**Fig. 2:**
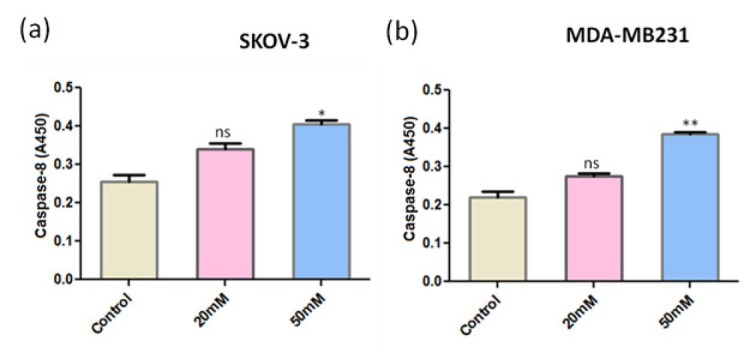
Expression of caspase-8 in SKOV-3 (a) and MDA-MB231 (b) cell lines upon treatment with ANDR for 4 h. Capsase-8 levels monitored by ELISA. Compared to the control: ns *P* >0.05, *P <0.05, **P < 0.01.

**Fig. 3:**
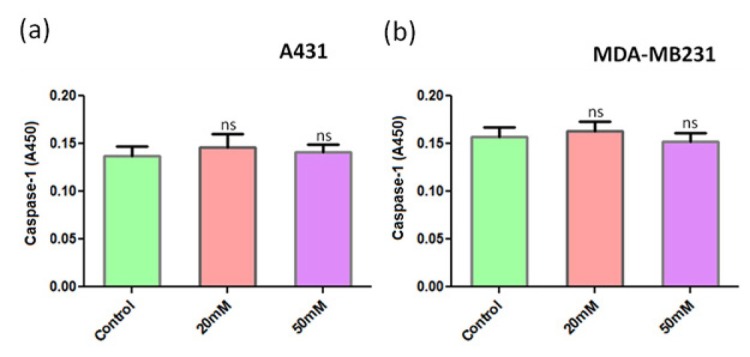
Expression of caspase-1 in A431 (a) and MDA-MB231 (b) cell lines upon treatment with ANDR for 4 h. Capsase-1 levels monitored by ELISA. Compared to the control: ns represents *P* >0.05, or no significant differences.

As ANDR inhibits the expression and activation of NFkB, which is essential for the inhibition of caspase activity, we explored the expression levels of caspase-8, an initiator caspase after ANDR treatment in breast and ovarian cancer cell lines MDA-MB231 and SKOV-3 by ELISA. We observed caspase-8 induction with increasing concentrations of ANDR in both breast and ovarian cancer cell lines (Fig. 2a and b). As ANDR is reported to stimulate an inflammatory response, we assessed the response of caspase-1 on ANDR treatment in ovarian and breast cancer cell lines (MDA-MB231 and SKOV-3, respectively) using ELISA. The results showed that there was no significant induction of caspase-1 upon ANDR treatment (Fig. 3a and b).

We found that ANDR inhibited NFkB signaling in the different cancer cell lines used in the present study, and as NFkB inhibits apoptosis by inducing the expression of FLIP (FLICE-like inhibitory protein) and XIAP (X-linked inhibitor of apoptosis protein), inhibitors of caspase-8 and apoptosis, respectively [35], we expected that the inhibitory effects of ANDR on NFkB activation might alleviate caspase-8 from its inhibitor FLIP. Our results showed that caspase-8 induction was observed with increasing concentrations of ANDR, whereas no significant increase in caspase-1 was observed. Because caspase-1 is known to initiate pyroptosis, which is an inflammatory lytic pathway [36], we concluded that ANDR may kill breast and ovarian cancer cells in a programmed apoptotic pathway rather than through pyroptosis that involves sudden lysis. This mechanism of induction of programmed cell death renders ANDR as an ideal anticancer drug because it would specifically kill cancer cells without harming normal cells.

### 3.3 Effect of ANDR on extra cellular matrix (ECM)

**Fig. 4:**
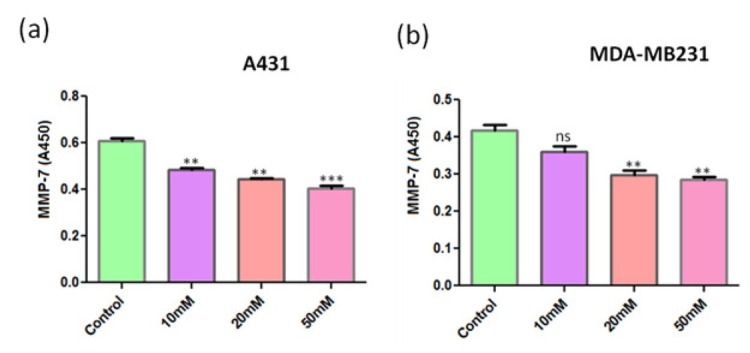
MMP-7 expression in A431 (a) and MDA-MB231 (b) cell lines upon treatment with increasing concentrations of ANDR for 4 h. MMP-7 levels monitored by ELISA. Compared to the control: ns >0.05, **P < 0.01, ***P < 0.001.

**Fig. 5:**
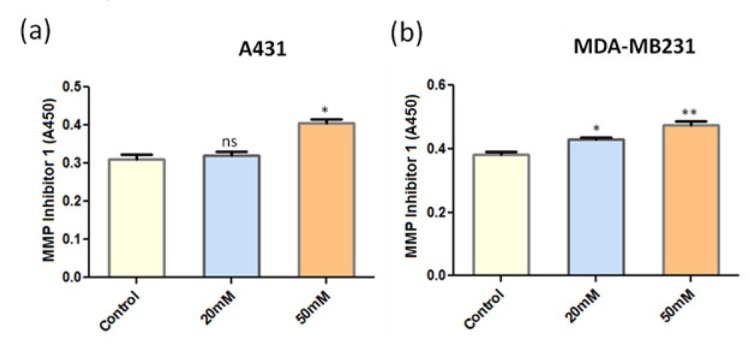
MMP inhibitor 1 expression in A431 (a) and MDA-MB231 (b) cell lines upon treatment with increasing concentrations of ANDR for 4 h. MMP Inhibitor 1 levels monitored by ELISA. Values shown are the average of triplicate measurements. Compared to the control: ns >0.05, *P < 0.05, **P < 0.01.

Using ELISA, we determined the effect of ANDR on the expression of MMP-7 in epidermoid carcinoma and breast cancer cell lines, A431 and MDA-MB231, respectively. We observed that MMP-7 levels were decreased in both the cancer cell lines upon increasing the concentration of ANDR (Fig. 4a and b). Due to the fact that TIMP (tissue inhibitors of metallopeptidases-1) is a metallopeptidase inhibitor that is predicted to interact with several other MMPs (Supplementary Figure 1), we further checked the expression levels of metallopeptidase inhibitor 1, which inhibits the activity of MMPs in epidermoid carcinoma and breast cancer cell lines, A431 and MDA-MB231, using ELISA. We observed an increase in the expression of metallopeptidase inhibitor 1 in correlation with ANDR treatment (Fig. 5a and b). Therefore, ANDR prevents the degradation of ECM by inhibiting MMPs and inducing metallopeptidase inhibitor 1. Hence, ANDR might inhibit metastasis of cancer cells.

It is known that cancer cells undergo metastasis once they are detached from the primary tumor site.

Angiogenesis is one such mechanism exhibited by cancer cells that leads to tumor development and progression [37]. Angiogenesis requires ECM remodeling that involves degradation by MMPs in the first stage [38]. MMP-7 or matrilysin is one such MMP that cleaves the macromolecules of ECM such as casein, aggrecan, laminin, gelatins, fibronectin, entactin, elastin, decorin, collagen IV, and tenascin casein [39, 40]. Hence, MMP-7 has been found to be highly expressed in gastrointestinal, ovarian, lung, and colorectal cancer cells, and it enhances tumor proliferation and metastasis [41, 42, 43]. Fortunately, cells have endogenous inhibitors for MMPs, called TIMPs that prevent metastasis [44]. In the present study, we analyzed the levels of MMP-7 and its inhibitor and found that ANDR treatment inhibited the expression of MMP-7 by stimulating the expression of metallopeptidase inhibitor 1, which in turn inhibited the activity of MMP-7.

Currently, a large number of drugs are under investigation, because they have the ability to inhibit the migration and invasion of cancer cells through the down-regulation of MMP-7; however, their use in clinical practice has been limited because of their side effects [45]. Among these, triptolide can be used for ovarian cancer treatment [46], but many studies have demonstrated that this drug has toxic effects on the female reproductive system [45, 47]. Therefore, our results suggest that ANDR is a promising candidate for development as a drug to treat both breast and ovarian cancers with minor side effects.

## 4 Conclusion

**Fig. 6:**
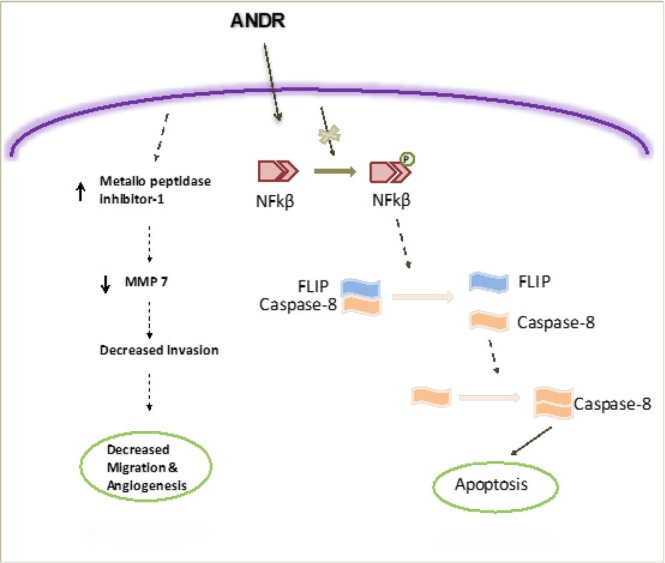
Schematic representation of the mechanism of ANDR effects on cancer cell lines. ANDR affects various signaling events leading to apoptosis and decreased migration and invasion.

We found that ANDR treatment could be used to prevent tumor invasion and migration (Fig. 6). This effect is achieved by the inhibition of NFkB activation that leads to caspase-8 induction. Such induction of caspase-8-mediated apoptosis has further implications in promoting the activity of metallopeptidase inhibitor 1, which in turn down-regulates the activity of MMP-7. Thus, this study suggests that with specific structural modifications to improve its efficacy, ANDR could emerge as a promising candidate in the development of a chemotherapeutic agent for breast and ovarian cancers.
