# Supplementary material for: Andrographolide as a Therapeutic Agent Against Breast and Ovarian Cancers
Source: Open Life Sci. 2019 Dec 4;14:462–9. doi: 10.1515/biol-2019-0052 (PMC7874781; doi:10.1515/biol-2019-0052)
Supplement: Supplementary file 1 [file biol-14-462_sm.pdf]

Supplementary Fig. 1: STRING analysis of TIMP-1

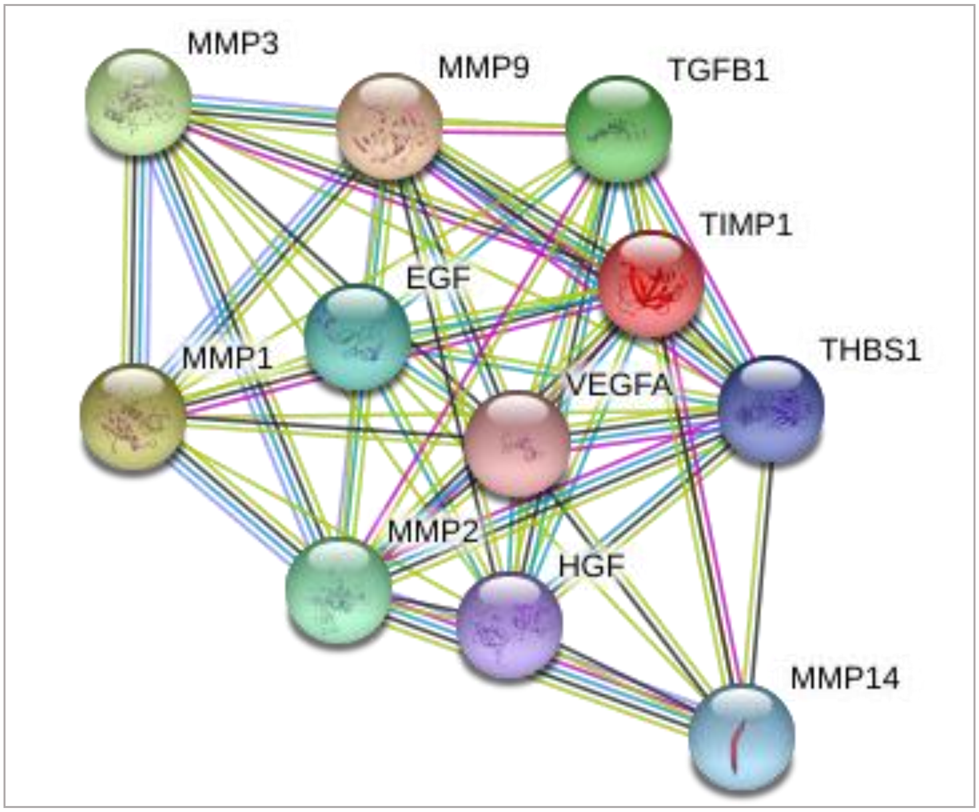

Supplementary Table 1:

Evaluation of cytotoxic effect of ANDR in Cancer Cell lines

Cancer Type	Cell line	24hrs	Ref
Breast Cancer	MDA-MB-231	51.98 µM	(24)
	MCF-7	61.11 µM	(24)
	T-47D	118.5 µM	(24)
	MCF-10A	137.9 µM	(24)
	MDA-MB-435	51.3 µM	(24)
	MDA-MB-231	58.1 µM	(24)
Cervix Cancer	KB cells	1.5ug/ml	(31)
	Hela	>40 µM	(29)
Colon Cancer	SW620	11 µM	(30)
	HCT-116	>40 µM	(29)
Gastric Cancer	SGC7901	42.7 µM	
Liver Cancer	Huh-7	17.6µg/ml	(25)
	SMMC-7721	78.7 µM	(26)
Leukemia	Jurkat E6-1	>100µM	(28)
	K562	30.2 µM	
	p388	1.0ug/ml	(31)
Lung Cancer	H522	16µM	(30)
	A549	>40 µM	(29)
Melanoma	M-14	14 µM	(30)
Ovarian Cancer	SKOV-3	28 µM	(30)
Prostate Cancer	DU145	28 µM	(30)
	PC-3	23.3 µM	(27)
Pancreas Cancer	MiaPaCa-2	>40 µM	(29)
Skin Cancer	A375	>40 µM	(29)
	A431	>50 µM	(32,33)
